# Effect of cocoa flavanol supplementation for the prevention of cardiovascular disease events: the COcoa Supplement and Multivitamin Outcomes Study (COSMOS) randomized clinical trial

**DOI:** 10.1093/ajcn/nqac055

**Published:** 2022-03-16

**Authors:** Howard D Sesso, JoAnn E Manson, Aaron K Aragaki, Pamela M Rist, Lisa G Johnson, Georgina Friedenberg, Trisha Copeland, Allison Clar, Samia Mora, M Vinayaga Moorthy, Ara Sarkissian, William R Carrick, Garnet L Anderson, COSMOS Research Group, JoAnn E Manson, Howard D Sesso, Pamela M Rist, Susanne Rautiainen Lagerstrom, Shari S Bassuk, Lu Wang, Aditi Hazra, Heike Gibson, Meryl S LeBoff, Samia Mora, Olivia I Okereke, Deirdre K Tobias, Nancy R Cook, Paulette D Chandler, William Christen, Georgina Friedenberg, Trisha Copeland, Jasmah Hanna, Allison Clar, Denise D'Agostino, Manickavasagar Vinayagamoorthy, Heike Gibson, Eunjung Kim, Martin Van Denburgh, Gregory Kotler, Chunying Li, Vadim Bubes, Ara Sarkissian, Doug Smith, Eduardo C Pereira, Melvyn Okeke, Elise Roche, David Bates, Claire Ridge, Alexandra Phillips, Brielle Salvo, Annalee Wilson, Leah Hall, Jimaldy Baez, Young-Hwan Sim, Hayara Cardoso, Gabriel Senor, Connor Rudnicki, Hanh Huynh, Viviane Nguyen, Nicholas Terrell, Beth A Holman, Joseph Walter, Lisa Fields Johnson, Amy Casarella, Julia O'Connell, William Christen, Susanne Rautiainen Lagerstrom, Luc Djoussé, Paulette D Chandler, Aditi Hazra, Deidre K Tobias, Zareen M Farukhi, Lu Wang, Xuehong Zhang, Kenneth Breen, George V Menjin Jr, Rolando Rodriguez, Shamikhah Curry, Samia Mora, Leah Arsenault, Olubunmi Solano, Alison Weinberg, Jennifer Coates, Matthew Kilroe, Lincoln Zernicke, Katelyn Hasson, Karen Matthew, Samia Mora, Chris Pfeffer, Julie Duszlak, David Bates, Vincent Guzman, Josue Falcon, Alex Romero, Henry Kupets, Frank Cortez, James C LeSuer, Andrea Hrbek, Eileen Bowes, Philomena Quinn, Megan Mele, Garnet L Anderson, Lisa Johnson, Leslie F Tinker, Aaron K Aragaki, Megan Herndon, Sue L Mann, Mary Pettinger, Rebecca P Hunt, Bill Carrick, Kate Szyperski, Lori Proulx-Burns, Elizabeth Burrows, Marian Limacher, Judith Hsia, Ganesh Asaithambi, Muhib Khan, Nandakumar Nagaraja, Lenore C Ocava, Jana Wold, Brian Silver, Stephanie Connelly, Gretchen Van Lom, Cris Garvida, Kathy Hightower, Patricia Spaulding, Wei Lin, Jenny Schoenberg, Patti Olee, Lawrence S Cohen, Theodore Colton, I Craig Henderson, Stephen Hulley, Alice H Lichtenstein, Eugene R Passamani, Rebecca A Silliman, Nanette Wenger, Shari E Ludlam, Hagen Schroeter, Michael Fare, Javier Ottawani, Catherine Kwik-Uribe, Cassandra Arnaiz, Ann Costanza, John Greene, Paul Hennessey, Sarma Vadlamani, Mallik Karmsetty, Paul Martini, Jan-Willem van Klinken, Alpa Shah, Lori Stern

**Affiliations:** Division of Preventive Medicine, Brigham and Women's Hospital and Harvard Medical School, Boston, MA, USA; Department of Epidemiology, Harvard T.H. Chan School of Public Health, Boston, MA, USA; Division of Preventive Medicine, Brigham and Women's Hospital and Harvard Medical School, Boston, MA, USA; Department of Epidemiology, Harvard T.H. Chan School of Public Health, Boston, MA, USA; Division of Public Health Sciences, Fred Hutchinson Cancer Research Center, Seattle, WA, USA; Division of Preventive Medicine, Brigham and Women's Hospital and Harvard Medical School, Boston, MA, USA; Department of Epidemiology, Harvard T.H. Chan School of Public Health, Boston, MA, USA; Division of Public Health Sciences, Fred Hutchinson Cancer Research Center, Seattle, WA, USA; Division of Preventive Medicine, Brigham and Women's Hospital and Harvard Medical School, Boston, MA, USA; Division of Preventive Medicine, Brigham and Women's Hospital and Harvard Medical School, Boston, MA, USA; Division of Preventive Medicine, Brigham and Women's Hospital and Harvard Medical School, Boston, MA, USA; Division of Preventive Medicine, Brigham and Women's Hospital and Harvard Medical School, Boston, MA, USA; Division of Cardiovascular Medicine, Brigham and Women's Hospital and Harvard Medical School, Boston, MA, USA; Division of Preventive Medicine, Brigham and Women's Hospital and Harvard Medical School, Boston, MA, USA; Division of Preventive Medicine, Brigham and Women's Hospital and Harvard Medical School, Boston, MA, USA; Division of Public Health Sciences, Fred Hutchinson Cancer Research Center, Seattle, WA, USA; Division of Public Health Sciences, Fred Hutchinson Cancer Research Center, Seattle, WA, USA

**Keywords:** cocoa extract, flavanols, cardiovascular disease, randomized clinical trial, cancer, multivitamin

## Abstract

**Background:**

Cocoa extract is a source of flavanols that favorably influence vascular risk factors in small and short-term trials, yet effects on clinical cardiovascular events are untested.

**Objectives:**

We examined whether cocoa extract supplementation decreases total cardiovascular disease (CVD) among older adults.

**Methods:**

We conducted a randomized, double-blind, placebo-controlled, 2-by-2 factorial trial of cocoa extract supplementation and multivitamins for prevention of CVD and cancer among 21,442 US adults (12,666 women aged ≥65 y and 8776 men aged ≥60 y), free of major CVD and recently diagnosed cancer. The intervention phase was June 2015 through December 2020. This article reports on the cocoa extract intervention. Participants were randomly assigned to a cocoa extract supplement [500 mg flavanols/d, including 80 mg (–)-epicatechin] or placebo. The primary outcome was a composite of confirmed incident total cardiovascular events, including myocardial infarction (MI), stroke, coronary revascularization, cardiovascular death, carotid artery disease, peripheral artery surgery, and unstable angina.

**Results:**

During a median follow-up of 3.6 y, 410 participants taking cocoa extract and 456 taking placebo had confirmed total cardiovascular events (HR: 0.90; 95% CI: 0.78, 1.02; *P* = 0.11). For secondary endpoints, HRs were 0.73 (95% CI: 0.54, 0.98) for CVD death, 0.87 (95% CI: 0.66, 1.16) for MI, 0.91 (95% CI: 0.70, 1.17) for stroke, 0.95 (95% CI: 0.77, 1.17) for coronary revascularization, neutral for other individual cardiovascular endpoints, and 0.89 (95% CI: 0.77, 1.03) for all-cause mortality. Per-protocol analyses censoring follow-up at nonadherence supported a lower risk of total cardiovascular events (HR: 0.85; 95% CI: 0.72, 0.99). There were no safety concerns.

**Conclusions:**

Cocoa extract supplementation did not significantly reduce total cardiovascular events among older adults but reduced CVD death by 27%. Potential reductions in total cardiovascular events were supported in per-protocol analyses. Additional research is warranted to clarify whether cocoa extract may reduce clinical cardiovascular events. This trial is registered at www.clinicaltrials.gov as NCT02422745.

See corresponding article on page 1501.

## Introduction

Cocoa is made from the bean of the cacao tree, *Theobroma cacao*, and has a long history of medicinal use (1) and potential health benefits based upon its flavanol and procyanidin content (2) also found in tea, grapes, wine, and other foods. Cocoa extract also contains methylxanthines such as theobromine and caffeine, which may enhance the vascular and central nervous system effects of cocoa flavanols (3, 4).

Prospective studies examining cocoa products restricted to chocolate intake (5–8) or to usual levels of dietary flavanol intake (9–12) with risk of cardiovascular disease (CVD) have been inconsistent, likely due to uncertainty of cocoa flavanol content and measurement error, although some meta-analyses suggest modest inverse associations with CVD (13, 14). Numerous short-term, small-scale dietary intervention studies have examined the cardiovascular effects of flavanols and procyanidins (15–17), which have included well-characterized cocoa and cocoa product test materials linked to cardiovascular (18, 19) and cardiometabolic (20) benefits. Data have shown improvements in endothelium-dependent vasodilation (21–24), blood pressure (BP) (21, 25–27), inflammation (28, 29), and platelet activation (30, 31), and provide insight into the absorption, distribution, metabolism, and excretion of flavanols (32–34) as well as cocoa's potential vascular effects due to intake of the flavanol (−)-epicatechin (24, 35).

However, no large-scale trials have evaluated flavanol-rich cocoa extract containing all potential bioactive components of the cocoa bean on clinical cardiovascular outcomes. We therefore initiated the COcoa Supplement and Multivitamin Outcomes Study (COSMOS), a pragmatic, large-scale, 2 × 2 factorial randomized trial testing a cocoa extract supplement and a typical multivitamin in the prevention of CVD and cancer among older women and men. This report focuses on the cocoa extract component of the trial.

## Methods

**FIGURE 1:**
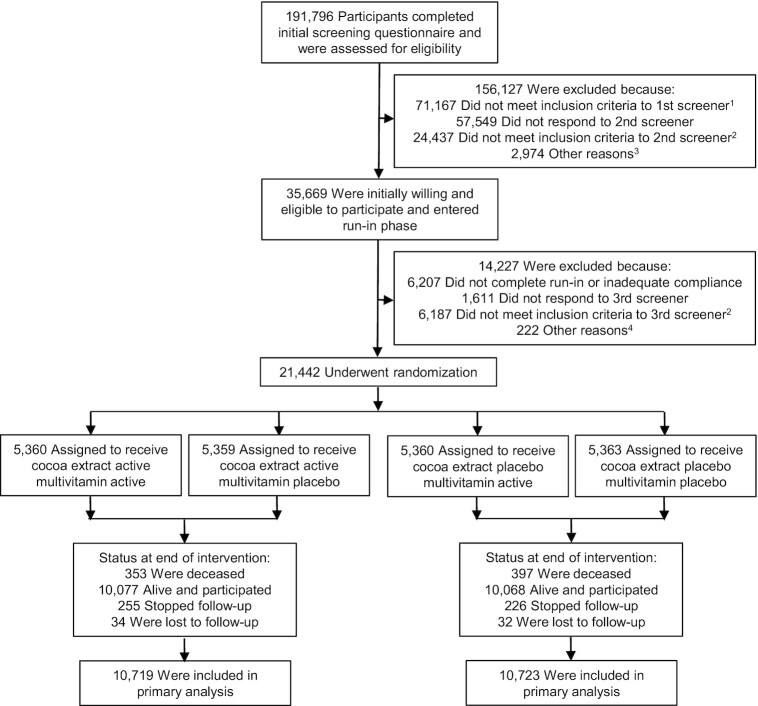
Screening, randomization, and follow-up of the participants. ^1^Eligibility was determined by medical history, age, and willingness to forego personal use of cocoa extract and multivitamin pills. ^2^Eligibility was determined by medical history, age, willingness to forego personal use of cocoa extract and multivitamin pills, caffeine sensitivity, and willingness to limit calcium and vitamin D supplement use. ^3^Included subjects who never completed the screening phase (*n* = 2914), eligibility could not be determined (*n* = 5), and enrollment goal already met (*n* = 55). ^4^Included subjects who never completed screening phase (*n* = 168) and enrollment goal already met (*n* = 54).

COSMOS (clinicaltrials.gov #NCT02422745) is a randomized, double-blind, placebo-controlled, 2 × 2 factorial trial testing a cocoa extract supplement [2 capsules/d containing 500 mg cocoa flavanols/d, including 80 mg (–)-epicatechin; supplied by Mars Edge] and a multivitamin supplement (Centrum Silver®; supplied by Pfizer Consumer Healthcare, now a part of GSK Consumer Healthcare) to prevent CVD and cancer in 21,442 US adults, including 12,666 women aged ≥65 y and 8776 men aged ≥60 y who were free of myocardial infarction (MI), stroke, and recently diagnosed cancer (except for nonmelanoma skin cancer) within the past 2 y (36). **Figure 1** summarizes the COSMOS trial design.

Recruitment included mailings to 71,521 active Women's Health Initiative (WHI) Extension Study participants (37) and mailings by Brigham and Women's Hospital (BWH) to 237,736 women and men contacted for, but not randomly assigned to, the VITamin D and OmegA-3 TriaL (VITAL) (38); mass mailings to 2,616,343 US men and women; and mailings to 3380 volunteers through other sources, including responses to media stories and advertisements. Of 191,796 participants completing a brief screening questionnaire, 120,629 were initially eligible and willing; then 63,025 completed another questionnaire with written informed consent before enrollment with oversight by the Human Subjects Committee at BWH. Participants were required to forego cocoa supplements (chocolate intake was not restricted) and multivitamins during the trial (vitamin D was limited to ≤1000 IU/d and calcium to ≤1200 mg/d from all supplements). Safety exclusions included renal failure or dialysis, cirrhosis, other serious conditions that precluded participation, and reported extreme sensitivity to caffeine, as the cocoa extract supplement had modest theobromine (∼50 mg/d) and caffeine (∼15 mg/d) content. A total of 35,669 eligible, willing, and consenting participants began at least a 2-mo placebo run-in to eliminate poor compliers (defined as taking <75% of the study pills) before randomization to increase study power (39). At the end of the run-in, participants returned a final compliance, eligibility, and risk factor questionnaire and semi-quantitative food-frequency questionnaire (40).

Baseline biospecimens were obtained during the run-in period from 6867 (32.0%) of 21,442 randomized participants, of whom 2050 participants comprised a longitudinal subcohort providing a baseline and at least 1 follow-up blood and/or spot urine sample at 1, 2, and/or 3 y follow-up. Mars Edge supported the measurement of the 5-(3′,4′-dihydroxyphenyl)-γ-valerolactone (gVL)-3′/4′-sulfate (gVL3S) and gVL-3′/4′-O-glucuronide (gVLG) metabolites (gVLM) (32), a biomarker of flavanol intake (41), to evaluate compliance with the cocoa extract intervention.

From April 2016 to March 2018, 21,442 participants meeting all eligibility criteria were randomized according to a schedule prepared and implemented by WHI staff to 1 of 4 arms in equal proportions: *1*) active cocoa extract and active multivitamin, *2*) active cocoa extract and multivitamin placebo, *3*) active multivitamin and cocoa extract placebo, or *4*) both placebos, using a computer-generated permuted block approach blinded to investigators and stratified by sex (women, men), age (separate 5-y age blocks for women and men), and recruitment source (WHI, BWH) in blocks of 12. Participants within a household were randomly assigned to the same intervention, when possible, to reduce cross-contamination risk. BWH staff sent randomized participants calendar packs containing cocoa extract or placebo capsules (and multivitamin or placebo tablets).

Participants were sent follow-up questionnaires, with calendar packs, at 6 mo and 12 mo following randomization and semi-annually thereafter, to assess compliance with randomized treatments (defined as taking ≥75% of the study pills), use of nontrial cocoa supplements and/or multivitamins, potential side effects of the interventions (refer to **Supplementary Figure 7**for the full list examined), updated medical history, and other relevant lifestyle, clinical, and dietary risk factors. COSMOS participants from the WHI received separate annual mailings from the WHI to update medical history and outcome follow-up. Loss to follow-up was greatly minimized via phone calls, e-mails, newsletters, communications with alternative contacts, and periodic National Death Index (NDI) searches. The trial was monitored by an external Data and Safety Monitoring Board. The randomized treatments continued through 31 December 2020, ending as scheduled, with a median (IQR) treatment period of 3.6 (3.2, 4.2) y. All participants provided written informed consent before enrollment in the trial, and trial activities were overseen by the Human Subjects Committee at BWH/Mass General Brigham.

### Primary and secondary trial outcomes

The primary outcome for the cocoa extract intervention was a composite total CVD outcome including incident MI, stroke, coronary revascularization, cardiovascular mortality, carotid artery surgery, peripheral artery surgery, and unstable angina requiring hospitalization. This was an expansion of our original primary CVD outcome definition of MI, stroke, coronary revascularization, and cardiovascular mortality because overall rates of CVD were lower than projected due to increasing use of statins and other preventive treatments, the influence of coronavirus disease 2019 (COVID-19) on 2020 CVD event rates, and a smaller proportion of older participants enrolled from WHI than anticipated. There were 14 secondary outcomes and 4 other outcomes that mostly consisted of subtypes of CVD or cancer. Secondary outcomes included a combination of total CVD and all-cause mortality, as well as individual components of total cardiovascular events, and the original composite CVD outcome. Secondary cancer outcomes included invasive cancer (excluding nonmelanoma skin cancer) key site-specific cancers including breast, colorectal, and lung cancer.

Participants reporting a primary or secondary study outcome signed a release form to request related medical records for evaluation and processing according to standardized WHI and BWH study procedures (42). Self-reported primary and secondary outcomes were confirmed by medical record review by a committee of physicians and investigators blinded to treatment assignment. MI (43), stroke (44, 45), and coronary revascularization, which included documented coronary artery bypass graft (CABG) surgery, percutaneous coronary intervention (PCI), coronary stent, or atherectomy (42), were confirmed using established criteria. Unstable angina requiring hospitalization included reports of increased pain, use of medications to alleviate pain, absence of evidence of MI, plus other related factors. Carotid artery surgery and peripheral artery surgery or revascularization included review and identification from surgical and radiology reports. Ischemic heart disease and stroke deaths were consistent with either outcome as an underlying cause. Incident cancers were confirmed by a pathology report that substantiated a malignant primary invasive cancer at any location other than nonmelanoma skin cancer (42); all histologic types and anatomic subsites were included. For participants determined to be deceased, we contacted next of kin to request permission to obtain medical records and a copy of the death certificate. For WHI participants, death certificates were alternatively requested from the state where the participant died. If records were unavailable or participants were lost to follow-up, we searched the NDI Plus for cause of death according to death certificate information. An end-points committee reviewed records to assign cause of death. Analyses included only confirmed outcomes.

### Statistical analyses

Our primary analyses were based on the intention-to-treat principle for time to first event data (46). Cox proportional hazards models estimated HRs using an indicator variable for cocoa extract treatment and stratifying the baseline hazard functions by sex, age group, recruitment source, and multivitamin intervention arm. COSMOS was designed for 22,000 participants with ≥80% power to detect a 11% relative hazard reduction in total CVD and >95% power to detect the same reduction for the secondary outcome of CVD plus all-cause mortality. We also had ≥90% power to detect a 14% reduction in total cancer. Models were constructed for each clinical outcome, where person-time for each outcome was counted as days from randomization to the first post-randomization diagnosis of the designated outcome. Follow-up was censored at date of last contact, death, or end of the trial on 31 December 2020, whichever came first.

Kaplan-Meier cumulative incidence curves, cumulative HRs, interactions between randomization groups with trial time, and analyses that excluded the first 1 and 2 y of follow-up assessed whether treatment effects varied over time (47). Nine subgroup analyses examined effect modification by a priori (concurrent multivitamin randomization assignment, sex, age, and use of statins or aspirin) and post hoc (history of CVD, smoking status, number of CVD risk factors, and chocolate consumption) factors. Secondary outcomes (*n* = 14) mostly constituted subtypes of CVD or cancer. Statistical significance (*P* ≤ 0.05) was assessed with 2-sided *P* values. We did not adjust *P* values or CIs for multiple testing. Consequently, results for secondary outcomes, other outcomes, subgroup analyses, and other analyses should be interpreted cautiously and considered hypothesis generating. At the nominal 0.05 level, we would expect <1 interaction and 1 secondary outcome to be significant by chance alone.

Per-protocol analyses censored follow-up when the participant discontinued trial pills, began outside nonstudy use of a cocoa supplement, and/or took <75% of study pills. HRs and 95% CIs were estimated using Cox regression models, weighted by the inverse probability of dependent censoring for noncompliance (48). Additional analyses compared self-reports of nonmonitored outcomes or potential side effects by intervention group. Analyses were performed using SAS version 9.4 (SAS Institute).

## Results

From June 2015 to March 2018 we randomly assigned 21,442 participants into COSMOS with a mean (±SD) age of 72.1 (±6.6) y. Women (mean age: 74.2 y) were older than men (mean age: 69.0 y), but sociodemographic, medical, and lifestyle factors at baseline were similar comparing the randomized cocoa extract and placebo groups (**Table 1** and **Supplementary Table 1**). Overall, there was a low proportion of current smokers (4.0%), nearly half took aspirin (48.9%), 42.1% took statins, 13.4% had diabetes, and 58.1% had high BP. A small subset of participants had a history of CVD or cancer at baseline (Table 1 and Supplementary Table 1). Few participants (0.4%) reported pre-enrollment cocoa extract supplement use.

**TABLE 1:** Characteristics of the participants at baseline, according to randomized assignment^1^

	Total (*n* = 21,442)		Cocoa extract (*n* = 10,719)		Placebo (*n* = 10,723)	
	*n*	(%)	*n*	(%)	*n*	(%)
Female sex	12,666	(59.1)	6337	(59.1)	6329	(59.0)
Age, mean ± SD, y	72.1 ± 6.6		72.1 ± 6.6		72.1 ± 6.6	
Hispanic/Latino^2^	544	(2.6)	252	(2.5)	292	(2.8)
Race/ethnicity^2^						
White	19,294	(90.0)	9624	(89.8)	9670	(90.2)
African American	1131	(5.3)	558	(5.2)	573	(5.3)
Asian/Pacific Islander	499	(2.3)	274	(2.6)	225	(2.1)
American Indian/Alaska Native	59	(0.3)	31	(0.3)	28	(0.3)
Multiracial/other/unknown or not reported	459	(2.1)	232	(2.2)	227	(2.1)
Education						
High school diploma/GED or less	2296	(10.8)	1141	(10.7)	1155	(10.9)
Attended or graduated from college	8685	(40.9)	4328	(40.8)	4357	(41.1)
Post-college	10,241	(48.3)	5147	(48.5)	5094	(48.0)
Smoking status						
Never	11,565	(54.7)	5766	(54.6)	5799	(54.9)
Past	8731	(41.3)	4396	(41.6)	4335	(41.0)
Current	835	(4.0)	398	(3.8)	437	(4.1)
Cocoa extract use before run-in	91	(0.4)	45	(0.4)	46	(0.4)
Chocolate consumption						
Monthly or less	6275	(31.8)	3095	(31.5)	3180	(32.2)
Weekly or daily	13,446	(68.2)	6740	(68.5)	6706	(67.8)
History of diabetes	2864	(13.4)	1417	(13.2)	1447	(13.5)
History of high blood pressure	12,423	(58.1)	6190	(57.9)	6233	(58.3)
Statin use	8911	(42.1)	4480	(42.3)	4431	(41.9)
Aspirin use	10,379	(48.9)	5211	(49.1)	5168	(48.7)
No. of cardiovascular risk factors^3^						
0–1	9159	(42.9)	4561	(42.7)	4598	(43.1)
2	6289	(29.5)	3167	(29.7)	3122	(29.3)
≥3	5901	(27.6)	2950	(27.6)	2951	(27.7)
History of cardiovascular disease^4^	1269	(6.0)	626	(5.9)	643	(6.1)
History of revascularization (CABG/PCI)	862	(4.0)	430	(4.0)	432	(4.0)
History of unstable angina	374	(1.8)	170	(1.6)	204	(1.9)
History of carotid artery surgery/stenting	93	(0.4)	47	(0.4)	46	(0.4)
History of peripheral artery surgery/stenting	144	(0.7)	75	(0.7)	69	(0.7)
History of heart failure	364	(1.7)	173	(1.6)	191	(1.8)
History of cancer excluding non-melanoma skin cancer	3550	(16.6)	1775	(16.6)	1775	(16.6)

1 *n* = 21,442. Percentages may not sum to 100 because of rounding. Data on age and sex were complete. Data on other characteristics, except for chocolate consumption, were available for ≥98.0% of the trial participants. Chocolate consumption was missing for 8.0% [*n* = 1721; 8.2% (*n* = 884) vs. 7.8% (*n* = 837)] of participants. CABG/PCI, coronary artery bypass graft and percutaneous coronary intervention; GED, General Educational Development.

2 Ethnic group and race were self-reported by participants. Multiracial participants self-identified with >1 race. Participants of other race or unknown race self-identified with those categories.

3 Cardiovascular risk factors were history of hypertension, diabetes, taking cholesterol-lowering medication, smoking (ever), and parental history of early myocardial infarction (<65 y).

4 Defined as history at baseline of CABG/PCI, unstable angina, carotid artery surgery/stenting, or peripheral artery surgery/stenting.

### Cocoa extract and total cardiovascular events

**FIGURE 2:**
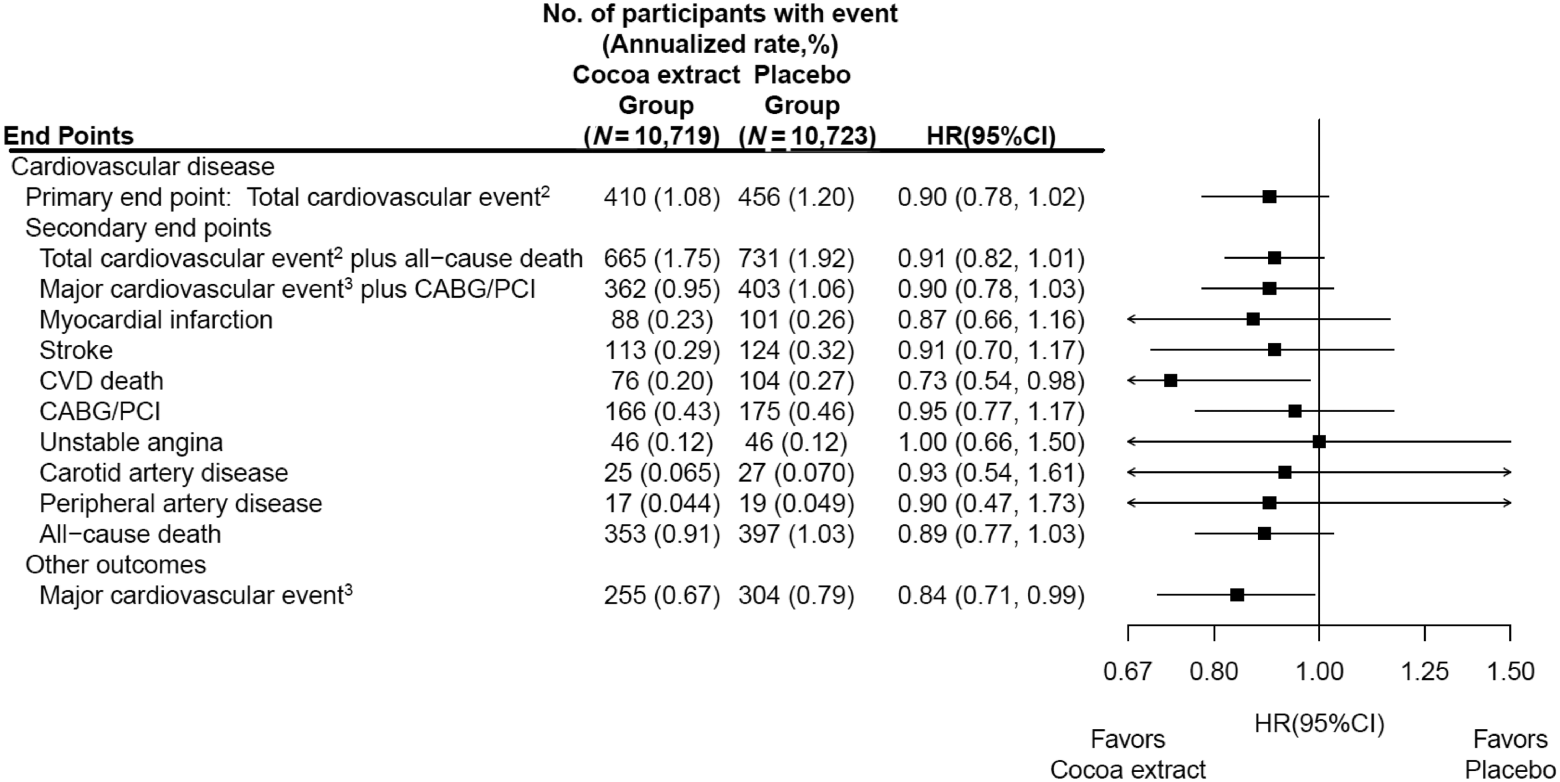
HRs and 95% CIs^1^ for the primary and secondary CVD outcomes, according to randomized assignment, in intention-to-treat analyses. ^1^Summary statistics were from Cox regression models that stratified baseline hazard functions by multivitamin trial randomization group, age, sex, and recruitment cohort. CIs were not adjusted for multiple comparisons. ^2^This outcome was a composite of myocardial infarction, stroke, CVD death, CABG/PCI, unstable angina including hospitalization, carotid artery surgery, and peripheral artery surgery. ^3^This outcome was a composite of myocardial infarction, stroke, and CVD death. CABG/PCI, coronary artery bypass graft and percutaneous coronary intervention; CVD, cardiovascular disease.

Median (IQR) follow-up of COSMOS participants through the study closeout on 31 December 2020 was 3.6 (3.2, 4.2) y, with 77,331 total person-years of follow-up. During the trial intervention period, 866 participants had confirmed total cardiovascular events, our primary outcome, including 189 first events of MI, 237 cases of stroke (194, 29, and 14 cases of ischemic, hemorrhagic, and unclassified stroke, respectively), 180 cases of cardiovascular death, 341 cases of coronary revascularization, 92 cases of unstable angina requiring hospitalization, 52 cases of carotid artery disease, and 36 cases of peripheral artery disease (**Figure 2**), with some participants experiencing multiple events. A total of 750 (3.5%) participants died during follow-up. The annualized rates of total cardiovascular events were 1.08% and 1.20% in the active and placebo cocoa extract groups, respectively.

Participants taking the cocoa extract supplement experienced no significant benefit on the primary outcome of total cardiovascular events (HR: 0.90; 95% CI: 0.78, 1.02) (Figure 2). This HR was identical to the original primary cardiovascular outcome of major cardiovascular events plus CABG/PCI (Figure 2). With regard to prespecified secondary outcomes, CVD death was significantly reduced (HR: 0.73; 95% CI: 0.54, 0.98), but no significant reductions were observed for MI, stroke, and revascularizations (Figure 2). No significant effect was seen for either ischemic or hemorrhagic stroke. For the prespecified secondary outcome of total CVD plus all-cause mortality, the HR (95% CI) was 0.91 (0.82, 1.01). Those randomly assigned to the cocoa extract supplement had a significant reduction in major cardiovascular events, a rigorous CVD outcome that included MI, stroke, and CVD death (HR: 0.84; 95% CI: 0.71, 0.99), although this was not a prespecified outcome. While there were fewer total deaths for those taking cocoa extract (353 deaths) compared with placebo (397 deaths), the mortality rates were not significantly different (HR: 0.89; 95% CI: 0.77, 1.03).

**FIGURE 3:**
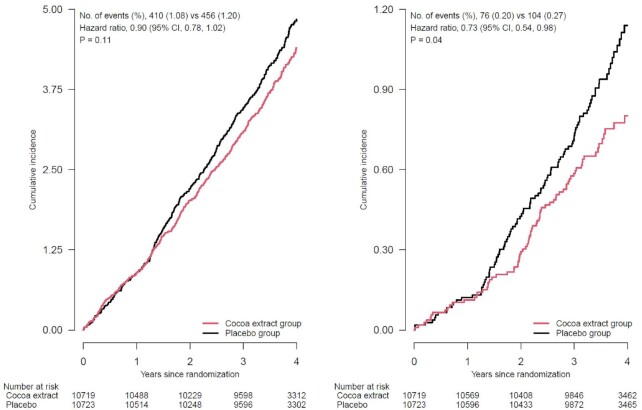
Cumulative incidence rates of total cardiovascular events^1^ and CVD death, according to year of follow-up, in the cocoa extract group and placebo group. ^1^Primary outcome (left panel): a composite of myocardial infarction, stroke, CVD death, CABG/PCI, unstable angina including hospitalization, carotid artery surgery, and peripheral artery surgery. Secondary outcome (right panel): CVD death. Summary statistics were from Cox regression models that stratified baseline hazard functions by multivitamin trial randomization group, age, sex, and recruitment cohort (intention-to-treat analyses). *P* value was for the effect of randomization group, based on a stratified score (log-rank) test. Rates (%) were annualized. CABG/PCI, coronary artery bypass graft and percutaneous coronary intervention; CVD, cardiovascular disease.

The cumulative incidence curves for the effect of cocoa extract compared with placebo on total cardiovascular events (log-rank *P* = 0.11) and CVD death (log-rank *P* = 0.04) (**Figure 3**) suggested a divergence in the hazard rates between treatment groups starting after 1 y of follow-up. Yet, latency analyses testing the effect of cocoa extract on total cardiovascular events beginning after year 1 (688 events) showed no significant reduction for total CVD (HR: 0.87; 95% CI: 0.75, 1.01). In **Supplementary Figure 1**, the cumulative HR generally stabilizes for total CVD after year 2 of follow-up. However, we found no violation of the proportional hazards assumption for any composite or individual cardiovascular outcomes.

**FIGURE 4:**
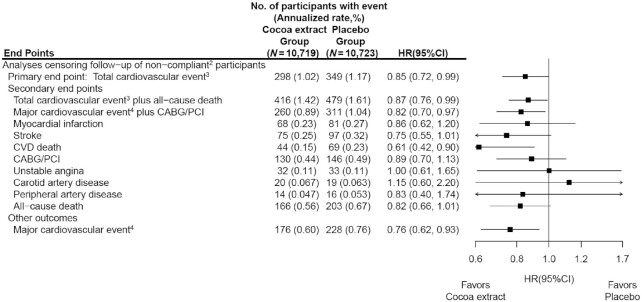
HRs and 95% CIs^1^ for the primary and secondary CVD outcomes, according to randomized assignment, where follow-up of noncompliant participants was censored. ^1^Summary statistics were from weighted Cox regression models that stratified baseline hazard functions by multivitamin trial randomization group, age, sex, and recruitment cohort, and used the robust sandwich estimator for variance. Time-dependent weights were calculated as the inverse probability of compliance, where probabilities were estimated from a Cox regression model with baseline hazard functions stratified by age, sex, recruitment source, multivitamin-trial arm and cocoa extract trial arm, and included baseline history of CVD and hypertension status (time-dependent) as covariates. CIs were not adjusted for multiple comparisons. ^2^A participant's follow-up was censored at the first time they reported having missed >8 d of cocoa extract study pills per month, took personal nonstudy cocoa extract, or did not respond to a semiannual questionnaire. ^3^Total cardiovascular event was a composite of myocardial infarction, stroke, CVD death, CABG/PCI, unstable angina including hospitalization, carotid artery surgery, and peripheral artery surgery. ^4^Major cardiovascular event was a composite of myocardial infarction, stroke, and CVD death. CABG/PCI, coronary artery bypass graft and percutaneous coronary intervention; CVD, cardiovascular disease.

Compliance (missing ≤8 d/mo of study pills) with active compared with placebo cocoa extract remained high at 88.6% and 89.3%, respectively, at 12 mo; 84.3% and 85.0% at 24 mo; 82.0% and 83.4% at 36 mo; and 83.1% and 84.2% at study closeout (**Supplementary Figure 2**). Less than 1% of participants began taking nonstudy cocoa extract supplements during the trial. Compliance with both pills and follow-up questionnaires resulted in corresponding compliance among those taking active compared with placebo cocoa extract at 36 mo (90.8% of randomized participants) of 78.1% and 79.3% (Supplementary Figure 2). History of CVD and hypertension, significant predictors of both dependent censoring and total CVD, was used to estimate weights for compliance-based analyses (48). In inverse probability–weighted Cox regression models for noncompliance defined in Supplementary Figure 2 (641 events), we found a corresponding HR (95% CI) for the primary outcome of total CVD events of 0.85 (0.72, 0.99) in per-protocol analyses censoring for nonadherence to study pills (for both the active and placebo groups) (**Figure 4**) compared with an HR of 0.90 (0.78, 1.02) in intention-to-treat analyses (Figure 2). The HRs for other CVD results were similarly strengthened (Figure 4).

In sensitivity analyses either allowing the baseline hazard to change on 1 January 2020 to account for the impact of the COVID-19 pandemic or accounting for intrahousehold correlation among 101 households with ≥2 participants randomly assigned to the same intervention arm using a robust sandwich estimator of variance (49), our results for total CVD were unchanged (HRs = 0.90) and were equal to our intention-to-treat analyses.

### Modifiers of the effect of cocoa extract on total CVD events

In subgroup analyses (**Supplementary Figure 3**), there was an apparent differential effect by ever smoking 100 or more cigarettes (*P-*interaction = 0.02); among ever-smokers the cocoa extract intervention reduced total CVD events, whereas among never-smokers there was no significant effect. We found no other evidence of effect modification by other baseline risk factors, including baseline history of CVD, or by the multivitamin intervention on total CVD (all *P*-interaction > 0.05).

### Cocoa extract and urinary gVLM concentrations

The influence of cocoa extract compared with placebo was confirmed via urinary gVLM concentrations assessed longitudinally at screening and 1, 2, and 3 y after randomization in 2050 participants (1060 active vs 990 placebo). Treatment with cocoa extract resulted in a more than 3-fold increase compared with placebo in gVLM concentrations, with an overall ratio of geometric means (95% CI) of 3.23 (2.84, 3.67; *P* < 0.001) that did not differ between follow-up assessments (*P* = 0.08) (**Supplementary Figure 4**).

### Cocoa extract and cancer

There were 1053 participants who had the secondary outcome of confirmed total invasive cancer. Participants taking cocoa extract experienced no significant effect on the secondary outcome of total cancer (HR: 1.10; 95% CI: 0.97, 1.24) (**Supplementary Figure 5**), which extended to prespecified site-specific cancers and cancer death.

### Potential adverse effects of cocoa extract supplementation

Cocoa extract had no significant effects on a range of nonmonitored cardiovascular, cancer, and other outcomes (all *P* > 0.05; **Supplementary Figure 6**). Among potential self-reported side effects associated with cocoa extract supplementation (Supplementary Figure 7), those taking the active cocoa extract supplement were 6% more likely to have nausea (HR: 1.06; 95% CI: 1.02, 1.11). In contrast, the cocoa extract group had significant 5% reductions in self-reported flulike symptoms and other headaches (both HRs: 0.95; 95% CI: 0.91, 0.99), as well as a larger 15% reduction in self-reported migraine (HR: 0.85; 95% CI: 0.78, 0.93).

## Discussion

COSMOS is the first large-scale, randomized, double-blind, placebo-controlled trial testing the long-term effects of cocoa flavanol supplementation in the prevention of CVD and cancer. We found that after a median of 3.6 y of treatment among older women and men with high compliance and minimal loss to follow-up, there was no statistically significant effect on the primary outcome of total cardiovascular events. However, cocoa flavanol supplementation significantly reduced CVD death by 27%, whereas other individual cardiovascular outcomes had no significant reductions in risk. In per-protocol analyses, cocoa extract reduced the primary outcome of total cardiovascular events among those compliant with the active intervention, compared with those compliant with the placebo. Finally, cocoa extract had no effect on the secondary outcomes of total invasive cancer and major site-specific cancers.

The potential cardiovascular benefits for cocoa extract have been supported by several smaller, short-term mechanistic trials among patients with and without coronary risk factors. These trials have provided broader insights on the absorption, metabolism, and excretion of flavanols in humans (33, 34), with those focused on cocoa flavanol intake (as beverages, supplements, or chocolate) at up to 2000 mg/d and up to 1 y of treatment. Meta-analyses support benefits for flavanols on cardiometabolic biomarkers including lipids and inflammation (20), endothelium-dependent vasodilation measured by flow-mediated dilation (23), BP (25), arterial stiffness measured by pulse-wave velocity (50), and insulin resistance (19). COSMOS is the first trial testing how these individual or combined cardiovascular mechanisms may translate into longer-term reductions in clinical cardiovascular events. We have longitudinal biospecimens and clinic-based assessments in a subcohort of participants to evaluate key cardiovascular mechanisms, including biomarkers, BP, and pulse-wave velocity, in future analyses.

Dietary flavonoids are a structurally diverse set of naturally occurring polyphenolic compounds in plant-based foods, and flavan-3-ols are derivatives of flavanols, a major subclass of flavonoids, that include complex, bioactive monomeric and polymeric compounds found in tea leaves, cocoa, grapes, and other foods (51). Self-reported diet in cohort studies is susceptible to measurement error, food and nutrient variability, and generalized nutrient-composition data (52). Nevertheless, prospective cohort studies have reported an inverse association between dietary flavanols—at lower concentrations than tested in COSMOS—with CVD (11, 12), carotid atherosclerosis (9), hypertension (10), and type 2 diabetes (14). A J-shaped association between chocolate intake and lower total and cardiovascular mortality was found in 91,891 participants (8). In the European Prospective Investigation into Cancer (EPIC)–Norfolk cohort, 15.6 g/d of chocolate intake compared with no intake was significantly associated with a 14% reduction in incident CVD (7) and a nonsignificant 13% reduction in heart failure (53). Residual confounding, however, limits observational studies examining flavanols or chocolate and CVD risk. Thus, our finding from COSMOS of suggestive cardiovascular benefits for cocoa extract would optimally be reproduced in subsequent trials to clarify any important potential public health implications for cocoa extract supplementation. This is supported by a significant 16% reduction in the more rigorous and clinically relevant—but not prespecified—composite outcome of major cardiovascular events (nonfatal MI, nonfatal stroke, and CVD death).

Cocoa extract was not significantly associated with all-cause mortality, but there was a significant 27% reduction in the secondary outcome of CVD mortality—the only secondary endpoint to reach nominal statistical significance—that may have reflected combined effects among individual CVD outcomes. There was an apparent split in the Kaplan-Meier curves for CVD death after year 1 and a reasonable biological rationale expected for atherosclerosis-related outcomes. However, we did not statistically confirm latency because of imprecision in our HR estimates from randomization to year 1 and the limited duration of the COSMOS trial intervention. We believe it is important to extend mortality follow-up to fully evaluate the long-term effects of cocoa extract on total and cause-specific mortality.

Cocoa extract had no effect on total and site-specific cancers. Several mechanisms have been proposed for flavanols on the inhibition of proliferation, inflammation, invasion, metastasis, and activation of apoptosis (54, 55). However, limited trials have tested cocoa on cancer mechanisms, with equivocal results (56, 57). Cohort studies are equally inconsistent, with some suggested benefits of flavanols on digestive cancers, including colorectal cancer (58) and gastric cancer in women but not in men (59). The short duration of follow-up limited our ability to evaluate cancer endpoints, given the long trial duration typically required for nutritional interventions to reduce cancer risk.

COSMOS was unique in testing cocoa extract, rather than just cocoa flavanols, against a placebo control. The 500 mg/d cocoa flavanols in COSMOS substantially exceeds the mean intake reported in Europe of 105 mg/d [ranging from 84 mg/d (Sweden) to 138 mg/d (Spain)] (60) and aligns with amounts tested in short-term trials (25, 61). Further, the COSMOS cocoa extract supplement avoided the perils of food-based cocoa interventions highly susceptible to variation in flavanol, theobromine, and other bioactive content (62). For example, cocoa pods collected from *T. cacao* are processed into nibs, cocoa liquor, cocoa butter, and cocoa powder, then alkalized to improve solubility, alter color, and modify flavor (63). The resulting variable changes in amino acids (64), polyphenols (65), methylxanthines including theobromine (66), and other functional compounds of cocoa products (63) contradict our goal to test a simple cocoa extract supplement that preserves the known bioactive contents of the cocoa bean without adding excessive sugar, saturated fat, and calories (67).

Strengths of COSMOS include the longest and only trial to date testing long-term cocoa extract supplementation on clinical events in a large older US population with high rates of follow-up and compliance based upon a 3-fold increase in associated gVLM concentrations and medical record–adjudicated primary and secondary outcomes. The cocoa extract intervention was well tolerated and taking it with food was recommended (68); the slightly greater likelihood of nausea likely reflects noncompliance with study guidelines. Complementary ancillary studies will examine cocoa extract supplementation on cognition, eye health, falls, physical performance, and other CVD- and aging-related outcomes.

Several potential limitations warrant consideration. First, our primary composite CVD endpoint included a wide mix of cardiovascular outcomes for sufficient power, whereas a narrower cardiovascular outcome limited to MI, stroke, and CVD death would have been more rigorous and biologically relevant. Second, we tested a cocoa extract supplement (**Supplementary Table 2**) containing all naturally occurring bioactive components of the cocoa bean, including cocoa flavanols, (–)-epicatechin, and theobromine, against a true placebo; thus, we cannot disentangle the effects of its individual components (69). Because we specifically tested 500 mg/d cocoa flavanols with 80 mg/d (–)-epicatechin, we could not evaluate other amounts for CVD risk. Third, we successfully leveraged the WHI, previously contacted potential VITAL trial participants, and mass mailings to expedite recruitment and randomization of 21,442 participants into COSMOS. Yet, generalizability may be limited, with modest racial and ethnic diversity and a volunteer bias for participants willing and eligible to enroll in a mail-based trial. Fourth, we conducted analyses of secondary and other outcomes and per-protocol and exploratory analyses for interactions and latency effects. Given the lack of effect for our primary outcome, these results warrant cautious interpretation as we did not account for multiple testing. Latency analyses excluded follow-up too early to be plausibly affected by the intervention. Fifth, the onset of the COVID-19 pandemic reduced CVD and cancer event rates in the final year of the COSMOS interventions and was partly responsible for our need to expand our primary cardiovascular outcome, although this ultimately made no difference on our HRs. Finally, our per-protocol analyses estimated the effects of cocoa extract if all participants were ≥75% compliant with taking their pills. The apparent observed 15% reduction in total cardiovascular events should be interpreted with caution as this secondary analysis is based on additional assumptions.

In conclusion, cocoa extract supplementation did not significantly reduce our primary outcome of total cardiovascular events after 3.6 y of treatment and follow-up, but we found a statistically significant 27% reduction in the secondary outcome of CVD death and a potential reduction in total cardiovascular events in per-protocol analyses. Longer-term follow-up of the trial cohort and ongoing ancillary mechanistic studies in COSMOS may further elucidate the relation between cocoa extract supplementation and clinical cardiovascular events.

## Supplementary Material

nqac055_Supplemental_File

## Data Availability

The data set(s) will be de-identified prior to release for sharing. We will make the data and associated documentation available to users only under a data-sharing agreement. Details on the availability of the study data to other investigators will be on our study website at https://cosmostrial.org/.

## Notes

The COcoa Supplement and Multivitamin Outcomes Study (COSMOS) is supported by an investigator-initiated grant from Mars Edge, a segment of Mars dedicated to nutrition research and products, which included infrastructure support and the donation of study pills and packaging. Pfizer Consumer Healthcare (now part of GSK Consumer Healthcare) provided support through the partial provision of study pills and packaging. COSMOS is also supported in part by grants AG050657, AG071611, EY025623, and HL157665 from the National Institutes of Health, Bethesda, MD. The Women's Health Initiative (WHI) program is funded by the National Heart, Lung, and Blood Institute, National Institutes of Health, US Department of Health and Human Services through contracts 75N92021D00001, 75N92021D00002, 75N92021D00003, 75N92021D00004, and 75N92021D00005. Neither company had a role in the trial design or conduct, data collection (other than blinded assays supported by Mars Edge and completed independently), data analysis, or manuscript preparation or review.

Supplementary Figures 1–7 and Supplementary Tables 1 and 2 are available from the “Supplementary data” link in the online posting of the article and from the same link in the online table of contents at https://academic.oup.com/ajcn/.

HDS and JEM contributed equally as co–first authors.

COSMOS was approved and overseen by the Institutional Review Board of Brigham and Women's Hospital/Mass General Brigham. The COSMOS website is www.cosmostrial.org.

Abbreviations used: BP, blood pressure; BWH, Brigham and Women's Hospital; CABG, coronary artery bypass graft; COSMOS, COcoa Supplement and Multivitamin Outcomes Study; COVID-19, coronavirus disease 2019; CVD, cardiovascular disease; gVLM, 5-(3′,4′-dihydroxyphenyl)-γ-valerolactone metabolite; MI, myocardial infarction; NDI, National Death Index; PCI, percutaneous coronary intervention; VITAL, VITamin D and OmegA-3 TriaL; WHI, Women's Health Initiative.
