# Supplementary material for: Effect of cocoa flavanol supplementation for the prevention of cardiovascular disease events: the COcoa Supplement and Multivitamin Outcomes Study (COSMOS) randomized clinical trial
Source: Am J Clin Nutr. 2022 Mar 16;115(6):1490–500. doi: 10.1093/ajcn/nqac055 (PMC9170467; doi:10.1093/ajcn/nqac055)

**On-line Supplementary Material**

**Effect of Cocoa Flavanol Supplementation for Prevention of Cardiovascular Disease Events:**

**The COSMOS Randomized Clinical Trial**

Howard D. Sesso, ScD, MPH*,^1,2^ JoAnn E. Manson, MD, DrPH*,^1,2^ Aaron K. Aragaki, MS,^3^ Pamela M. Rist, ScD,^1,2^ Lisa G. Johnson, PhD,^3^ Georgina Friedenberg, MPH,^1^ Trisha Copeland, MS,^1^ Allison Clar, BS,^1^ Samia Mora, MD, MHS,^1,4^ M. Vinayaga Moorthy, PhD,^1^ Ara Sarkissian, MA,^1^ William R. Carrick, BS,^3^ and Garnet L. Anderson, PhD^3^ for the COSMOS Research Group

**Supplementary Table 1. Characteristics of the participants at baseline, according to randomized assignment (N = 21,442)^1^**

**Supplementary Table 2. Composition of the cocoa extract supplement tested in the COcoa Supplement and Multivitamin Outcomes Study (COSMOS).^1^**

**Supplementary Figure 1. Cumulative hazard ratios of total cardiovascular events^1^ and CVD death, according to year of follow-up, in cocoa extract group and placebo group.**

**^1^** Primary outcome (left panel); a composite of myocardial infarction, stroke, CVD death, coronary artery bypass graft and percutaneous coronary intervention (CABG/PCI), unstable angina including hospitalization, carotid artery surgery, and peripheral artery surgery. Secondary outcome (right panel); CVD death.

Summary statistics were from Cox regression models that stratified baseline hazard functions by multivitamin trial randomization group, age, sex and recruitment cohort (intention-to-treat analyses). P-value was for the effect of randomization group, based on a stratified score (log-rank) test. Rates (%) were annualized.

Cumulative HRs (95% CI) were computed under the proportional hazards assumption, using increasingly longer cumulative follow-up elapsed from randomization. For example, if the trial had ended after only 2 years of follow-up, the resulting HR (95% CI) = 0.92 (0.76, 1.11) for 213 vs 233 total cardiovascular events (left panel) and the blue reference line indicates the estimated HR = 0.90 for total cumulative follow-up.

**Supplementary Figure 2. Proportion of participants who reported abstaining from use of non-study cocoa supplements, compliance to study pills, total compliance (adherence to both components),^1^ and total compliance to both study-related pills and follow-up questionnaires**^2^ **during the intervention phase (N=21,442).**

Dot (whisker) plots summarize proportion of participants that reported compliance (95% CI) for each semi-annual questionnaire during follow-up. Only compliance to study pills was assessed at 18 months and close-out (red).

**^1^** Total compliance was defined as self-report of having missed ≤ 8 days of cocoa flavanol study pills per month and did not take personal non-study cocoa extract. Participants with total compliance were compliant for both components, so could not be computed at 18 months or at closeout. Due to missing data from either component, total compliance could not be ascertained on N = 165 (0.8%), 430 (2.1%), 898 (4.4%), 533 (2.9%), 770 (4.1%), 326 (3.7%) and 206 (3.4%) of respondents at 6, 12, 24, 30, 36, 42 and 48 months, respectively.

**^2^** Participants not known to be deceased and willing to complete questionnaires within 2 months of the corresponding time point.

**^3^** Use of personal non-study cocoa extract was not assessed at 18m or at closeout, so total compliance^1^ could not be computed either.

**^4^** Study closeout occurred on December 31, 2020, so length of time from randomization to close-out varied; median (Q1 – Q3) = 43.5 (39.7 – 50.3) months.

**Supplementary Figure 3. Hazard ratios^1^ of the primary outcome**^2^ **according to subgroup, comparing cocoa extract group with placebo group.**

**^1^** Summary statistics were from Cox regression models that stratified baseline hazard functions by multivitamin trial randomization group, age, sex, recruitment cohort and subgroup. P-value was for the interaction (product term) between randomization group and participant characteristic based on a stratified score (log-rank) test; 1–degree-of-freedom score tests for trend were used for age group and number of CVD risk factors. Analyses were not adjusted for multiple comparisons.

**^2^** This outcome was a composite of myocardial infarction, stroke, CVD death, coronary artery bypass graft and percutaneous coronary intervention (CABG/PCI), unstable angina including hospitalization, carotid artery surgery, and peripheral artery surgery.

**^3^** Defined as history at baseline of CABG/PCI, unstable angina, carotid artery surgery/stenting or peripheral artery surgery/stenting.

**^4^** Cardiovascular risk factors were history of hypertension, diabetes, taking cholesterol-lowing medication, smoking (ever), and parental history of early myocardial infarction (<65 years).

**^5^** Multivitamin-multimineral.

Participants were missing baseline data for history of CVD (n = 348; 1.6%), ever smoked (n = 311; 1.5%), no. of cardiovascular risk factors (n = 93; 0.4%) chocolate consumption (n = 1721; 8.0%), statin use (n = 268; 1.2%) and aspirin use (n = 206; 1.0%).

**Supplementary Figure 4. Comparison of geometric means and 95% confidence intervals of gVLM^1^ at baseline and 1, 2, and 3 years follow-up, comparing cocoa extract group with placebo group.**

**^1^** 5-(3′,4′-dihydroxyphenyl)-[gamma]-valerolactone metabolites (gVLM) are a biomarker of flavanol intake and the sum of two metabolites (5-(3′,4′-dihydroxyphenyl)-[gamma]-valerolactone (gVL)-3′/4′-sulphate (gVL3S) and gVL-3′/4′-O-glucuronide (gVLG)) measured in the longitudinal biospecimen subcohort. There were 2,050 (1,060 in the cocoa extract group versus 990 in the placebo group) participants with a baseline and at least one follow-up measurement at 1, 2, and 3 years follow-up.

**Supplementary Figure 5. Hazard ratios and 95% confidence intervals^1^ for secondary cancer outcomes, according to randomized assignment, in intention-to-treat analyses.**

**^1^** Summary statistics were from Cox regression models that stratified baseline hazard functions by multivitamin trial randomization group, age, sex, and recruitment cohort. Confidence intervals were not adjusted for multiple comparisons.

**^2^** Total invasive cancer was a composite of invasive cancers of any site other than non-melanoma skin cancer.

**Supplementary Figure 6. Hazard ratios and 95% confidence intervals^1^ for self-reported non-monitored outcomes^2^ according to randomized assignment, in intention-to-treat analyses.**

**^1^** Summary statistics were from Cox regression models that stratified baseline hazard functions by multivitamin trial randomization group, age, sex and recruitment cohort. Analyses were not adjusted for multiple comparisons.

**^2^** Collected from all COSMOS trial participants at 12, 24, 36, 48 months and study closeout; self-reports from WHI participants were augmented with WHI data. Participants were asked to report only new diagnoses within the last year. Does not exclude participants that may have reported a prevalent condition at baseline.

**^3^** Not collected at 12 months. Summary statistics adjusted for the shorter follow-up.

**Supplementary Figure 7. Hazard ratios and 95% confidence intervals^1^ for side effects^2^ according to randomized assignment, in intention-to-treat analyses.**

**^1^** Summary statistics were from Cox regression models that stratified baseline hazard functions by multivitamin trial randomization group, age, sex and recruitment cohort. Analyses were not adjusted for multiple comparisons.

**^2^** Reports at 6, 18, 30 and 42 months reflect symptoms experienced in the last 6 months, while reports at 12, 24, 36, 48 months and study closeout reflect symptoms experienced over the past year.

**Supplementary Table 1. Characteristics of the participants at baseline, according to randomized assignment (N = 21,442)^1^**

|  | **Total**  **(N = 21,442)** | | **Cocoa extract**  **(N = 10,719)** | | **Placebo**  **(N = 10,723)** | |
| --- | --- | --- | --- | --- | --- | --- |
|  | **N** | **%** | **N** | **%** | **N** | **%** |
| Female sex – no. (%) | 12666 | (59.1) | 6337 | (59.1) | 6329 | (59.0) |
| Age – y | 72.1 | ±6.6 | 72.1 | ±6.6 | 72.1 | ±6.6 |
| Age group, y – no. (%) |  |  |  |  |  |  |
| 60 – 64 | 2705 | (12.6) | 1351 | (12.6) | 1354 | (12.6) |
| 65 – 69 | 6519 | (30.4) | 3259 | (30.4) | 3260 | (30.4) |
| 70 – 74 | 5774 | (26.9) | 2886 | (26.9) | 2888 | (26.9) |
| 75 – 79 | 3751 | (17.5) | 1876 | (17.5) | 1875 | (17.5) |
| 80 – 84 | 1761 | (8.2) | 882 | (8.2) | 879 | (8.2) |
| 85 – 89 | 741 | (3.5) | 369 | (3.4) | 372 | (3.5) |
| ≥90 | 191 | (0.9) | 96 | (0.9) | 95 | (0.9) |
| Recruitment source – no. (%) |  |  |  |  |  |  |
| WHI | 4611 | (21.5) | 2304 | (21.5) | 2307 | (21.5) |
| VITAL | 6943 | (32.4) | 3459 | (32.3) | 3484 | (32.5) |
| Other | 9888 | (46.1) | 4956 | (46.2) | 4932 | (46.0) |
| Hispanic/Latino – no. (%) ^2^ | 544 | (2.6) | 252 | (2.5) | 292 | (2.8) |
| Race – no. (%) ^2^ |  |  |  |  |  |  |
| White | 19294 | (90.0) | 9624 | (89.8) | 9670 | (90.2) |
| African American | 1131 | (5.3) | 558 | (5.2) | 573 | (5.3) |
| Asian/Pacific Islander | 499 | (2.3) | 274 | (2.6) | 225 | (2.1) |
| American Indian/Alaska Native | 59 | (0.3) | 31 | (0.3) | 28 | (0.3) |
| Multiracial/other/unknown or not reported | 459 | (2.1) | 232 | (2.2) | 227 | (2.1) |
| Education – no. (%) |  |  |  |  |  |  |
| HS diploma/GED or less | 2296 | (10.8) | 1141 | (10.7) | 1155 | (10.9) |
| Attended or graduated from college | 8685 | (40.9) | 4328 | (40.8) | 4357 | (41.1) |
| Post-college | 10241 | (48.3) | 5147 | (48.5) | 5094 | (48.0) |
| Geographic region – no. (%) |  |  |  |  |  |  |
| Northeast^3^ | 5941 | (27.7) | 2945 | (27.5) | 2996 | (27.9) |
| Midwest/Mountain | 5071 | (23.6) | 2536 | (23.7) | 2535 | (23.6) |
| South | 6151 | (28.7) | 3064 | (28.6) | 3087 | (28.8) |
| West/Southwest | 4279 | (20.0) | 2174 | (20.3) | 2105 | (19.6) |
| Smoking status – no. (%) |  |  |  |  |  |  |
| Never | 11565 | (54.7) | 5766 | (54.6) | 5799 | (54.9) |
| Past | 8731 | (41.3) | 4396 | (41.6) | 4335 | (41.0) |
| Current | 835 | (4.0) | 398 | (3.8) | 437 | (4.1) |
| Total MET-hours per week from exercise– median [interquartile range] | 17.1 | [5.2- 32.8] | 17.3 | [5.1- 33.0] | 17.0 | [5.3- 32.6] |
| Body mass index, kg/m^2^ – median [interquartile range] ^4^ | 26.8 | [24.0- 30.4] | 26.7 | [24.0- 30.4] | 26.9 | [24.0- 30.4] |
| gVLM – μM, median (IQR) ^5^ | 3.5 | [0.8 – 11.9] | 3.6 | [0.8 – 12.0] | 3.4 | [0.8 – 11.7] |
| Body mass index, kg/m^2^ – no. (%) ^4^ |  |  |  |  |  |  |
| <25 | 7070 | (33.6) | 3584 | (34.1) | 3486 | (33.2) |
| 25 - <30 | 8230 | (39.2) | 4062 | (38.7) | 4168 | (39.6) |
| ≥30 | 5718 | (27.2) | 2859 | (27.2) | 2859 | (27.2) |
| Multivitamin use before run-in – no. (%) | 8795 | (41.2) | 4438 | (41.6) | 4357 | (40.8) |
| Cocoa extract use before run-in – no. (%) | 91 | (0.4) | 45 | (0.4) | 46 | (0.4) |
| Chocolate consumption – no. (%) |  |  |  |  |  |  |
| Monthly or less | 6275 | (31.8) | 3095 | (31.5) | 3180 | (32.2) |
| Weekly or daily | 13446 | (68.2) | 6740 | (68.5) | 6706 | (67.8) |
| Statin use – no. (%) | 8911 | (42.1) | 4480 | (42.3) | 4431 | (41.9) |
| Aspirin use – no. (%) | 10379 | (48.9) | 5211 | (49.1) | 5168 | (48.7) |
| NSAID use – no. (%) | 6143 | (29.0) | 3041 | (28.7) | 3102 | (29.3) |
| History of diabetes – no. (%) | 2864 | (13.4) | 1417 | (13.2) | 1447 | (13.5) |
| History of high blood pressure – no. (%) | 12423 | (58.1) | 6190 | (57.9) | 6233 | (58.3) |
| Current use of medication for high blood pressure – no. (%) | 11217 | (53.0) | 5575 | (52.7) | 5642 | (53.2) |
| Systolic blood pressure, mm Hg – no. (%)^4^ |  |  |  |  |  |  |
| <120 | 6049 | (32.4) | 3036 | (32.5) | 3013 | (32.4) |
| 120-139 | 10793 | (57.8) | 5364 | (57.4) | 5429 | (58.3) |
| ≥140 | 1817 | (9.7) | 949 | (10.2) | 868 | (9.3) |
| Diastolic blood pressure, mm Hg – no. (%)^4^ |  |  |  |  |  |  |
| <80 | 13708 | (73.8) | 6863 | (73.8) | 6845 | (73.8) |
| 80-89 | 4401 | (23.7) | 2199 | (23.6) | 2202 | (23.8) |
| ≥90 | 465 | (2.5) | 243 | (2.6) | 222 | (2.4) |
| No. of cardiovascular risk factors – no. (%) |  |  |  |  |  |  |
| 0 – 1 | 9159 | (42.9) | 4561 | (42.7) | 4598 | (43.1) |
| 2 | 6289 | (29.5) | 3167 | (29.7) | 3122 | (29.3) |
| ≥3 | 5901 | (27.6) | 2950 | (27.6) | 2951 | (27.7) |
| History of cardiovascular disease – no. (%) | 1269 | (6.0) | 626 | (5.9) | 643 | (6.1) |
| History of revascularization (CABG/PCI) – no. (%) | 862 | (4.0) | 430 | (4.0) | 432 | (4.0) |
| History of unstable angina – no. (%) | 374 | (1.8) | 170 | (1.6) | 204 | (1.9) |
| History of carotid artery surgery/stenting – no. (%) | 93 | (0.4) | 47 | (0.4) | 46 | (0.4) |
| History of peripheral artery surgery/stenting – no. (%) | 144 | (0.7) | 75 | (0.7) | 69 | (0.7) |
| History of heart failure – no. (%) | 364 | (1.7) | 173 | (1.6) | 191 | (1.8) |
| History of cancer excluding non-melanoma skin cancer – no. (%) | 3550 | (16.6) | 1775 | (16.6) | 1775 | (16.6) |
| History of macular degeneration – no. (%) | 623 | (3.0) | 301 | (2.9) | 322 | (3.1) |
| History of cataract – no. (%) | 8629 | (40.8) | 4266 | (40.3) | 4363 | (41.3) |
| History of cataract surgery – no. (%) | 6413 | (30.2) | 3186 | (30.0) | 3227 | (30.5) |

^1^ Plus–minus values are means ± standard deviations. Medians [interquartile range] summarize skewed variables. Percentages may not sum to 100 because of rounding. Data on age and sex were complete.

^2^ Ethnic group and race were self-reported by participants. Multiracial participants self-identified with more than one race. Participants of other race or unknown race self-identified with those categories.

^3^ Includes one individual living on a US Army Base in Europe.

^4^ Calculated from self-report.

^5^ The 5-(3′,4′-dihydroxyphenyl)-[gamma]-valerolactone (gVL)-3′/4′-sulphate (gVL3S) and gVL-3′/4′-O-glucuronide (gVLG) metabolites (gVLM) is a biomarker of flavanol intake. gVLM is the sum of two metabolites (gVL3S and gVL3G) measured in the longitudinal biospecimen subcohort. There were 2,050 (1,060 in the cocoa extract group versus 990 in the placebo group) participants with a baseline and at least one follow-up measurement at 1, 2, and 3 years follow-up.

**Supplementary Table 2. Composition of the cocoa extract supplement tested in the COcoa Supplement and Multivitamin Outcomes Study (COSMOS).^1^**

|  | Cocoa extract supplement (2 capsules/day) |
| --- | --- |
| Total cocoa flavanols (DP 1-7) mg ^2^ | 500 ± 50 |
| Total flavanol monomers ^3^ | 110 ± 10 |
| (-)-epicatechin, mg | 80 ± 10 |
| (+)-catechin, mg | 3 ± 2 |
| (-)-catechin, mg | 25 ± 5 |
| Calories, kcal | <5 |
| Total fat, g | <0.2 |
| Saturated fat, g | <0.15 |
| Total carbohydrates, g | <0.5 |
| Sugars, g | 0 |
| Protein, g | <0.1 |
| Fiber, g | <0.5 |
| Theobromine, mg | 50 ± 5 |
| Caffeine, mg | 15 ± 5 |

^1^ Plus–minus values are means ± SD. Test products are not a significant source (≤1 mg/serving) of sodium, potassium, iron, magnesium, copper, manganese, phosphorous, or calcium.

^2^ Analysis based on AOAC 2020.05. Degrees of polymerization (DP) 1-7 includes flavanol monomers (DP 1) as well as procyanidins with up to 7 flavanol-units (DP 2-7).

^3^ Analysis based on AOAC 2013.04.

**Supplementary Figure 1. Cumulative hazard ratios of total cardiovascular events^1^ and CVD death, according to year of follow-up, in cocoa extract group and placebo group (N = 21,442).**


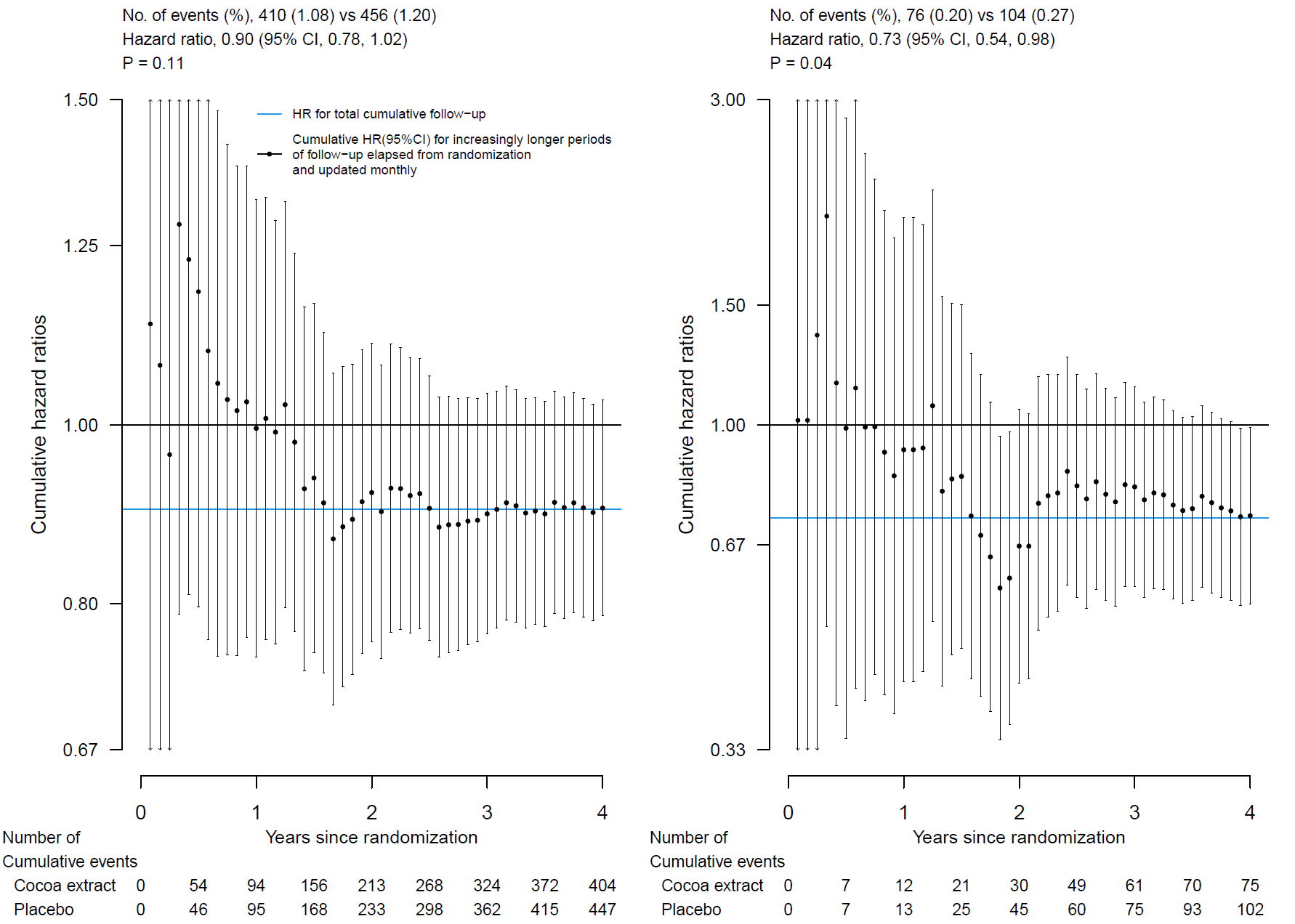


**Supplementary Figure 2. Proportion of participants who reported abstaining from use of non-study cocoa supplements, compliance to study pills, total compliance (adherence to both components),^1^ and total compliance to both study-related pills and follow-up questionnaires**^2^ **during the intervention phase (N=21,442).**


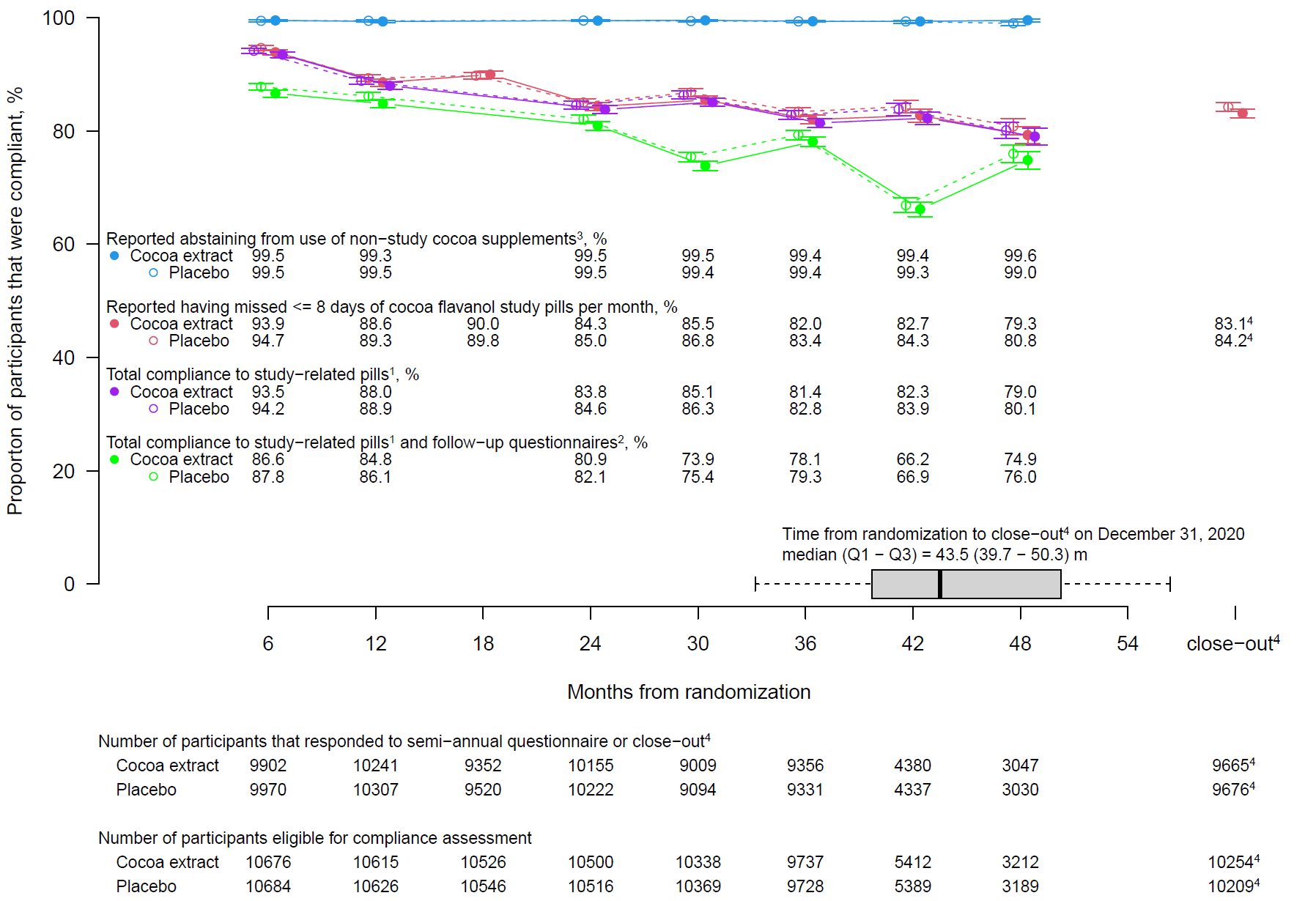


**Supplementary Figure 3. Hazard ratios^1^ of the primary outcome**^2^ **according to subgroup, comparing cocoa extract group with placebo group.**


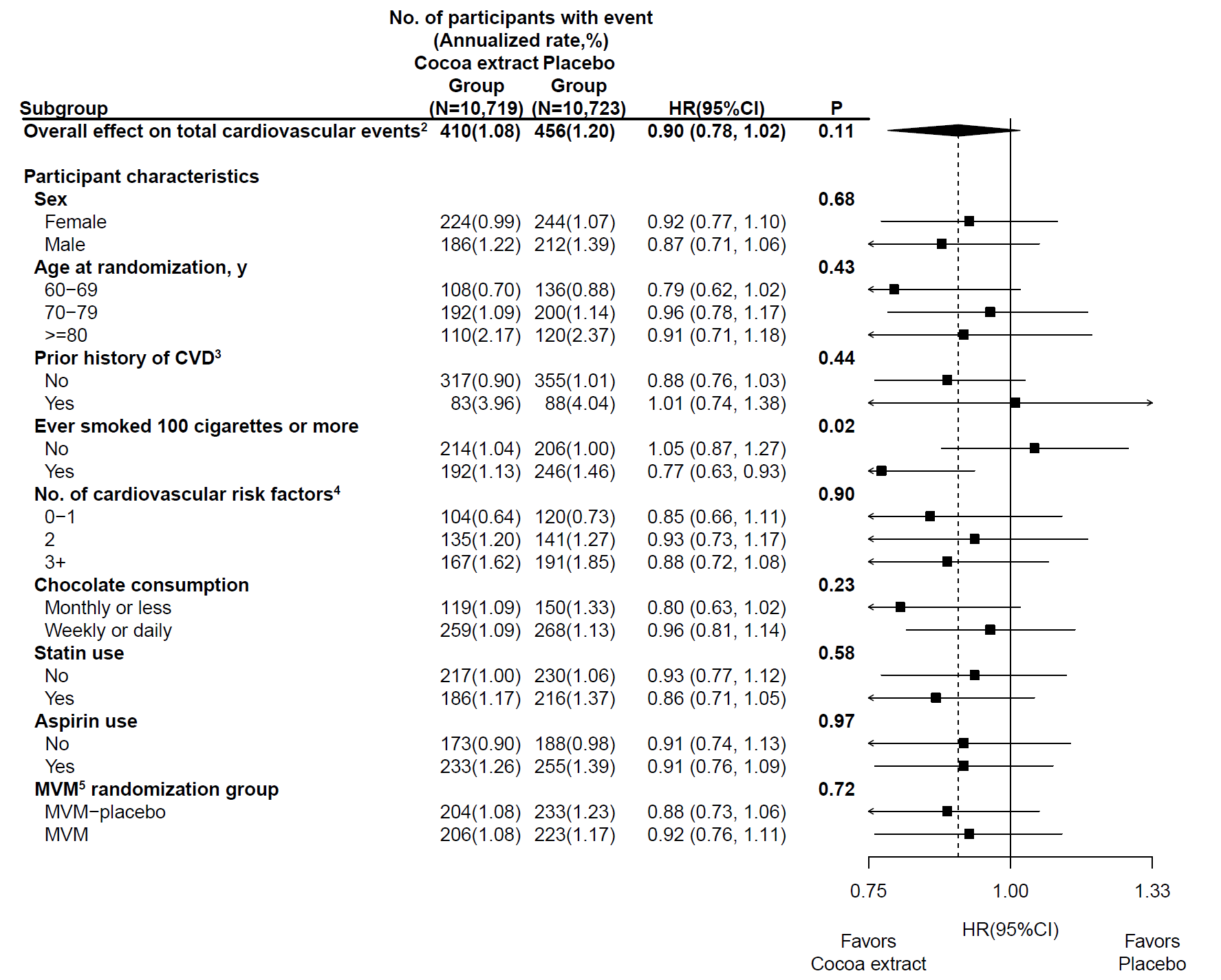


**Supplementary Figure 4. Comparison of geometric means and 95% confidence intervals of gVLM^1^ at baseline and 1, 2, and 3 years follow-up, comparing cocoa extract group with placebo group^2^.**


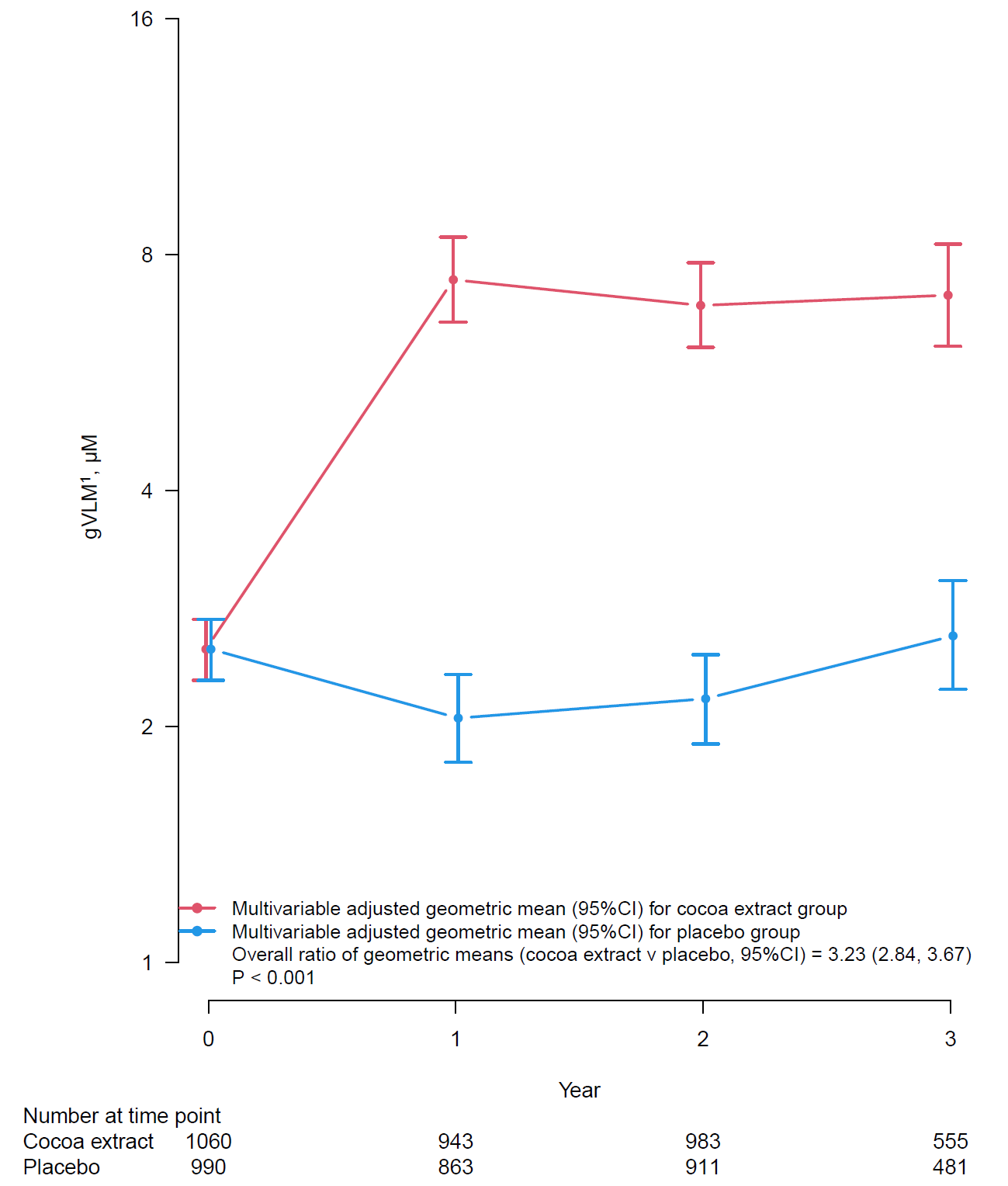


**Supplementary Figure 5. Hazard ratios and 95% confidence intervals^1^ for secondary cancer outcomes, according to randomized assignment, in intention-to-treat analyses.**


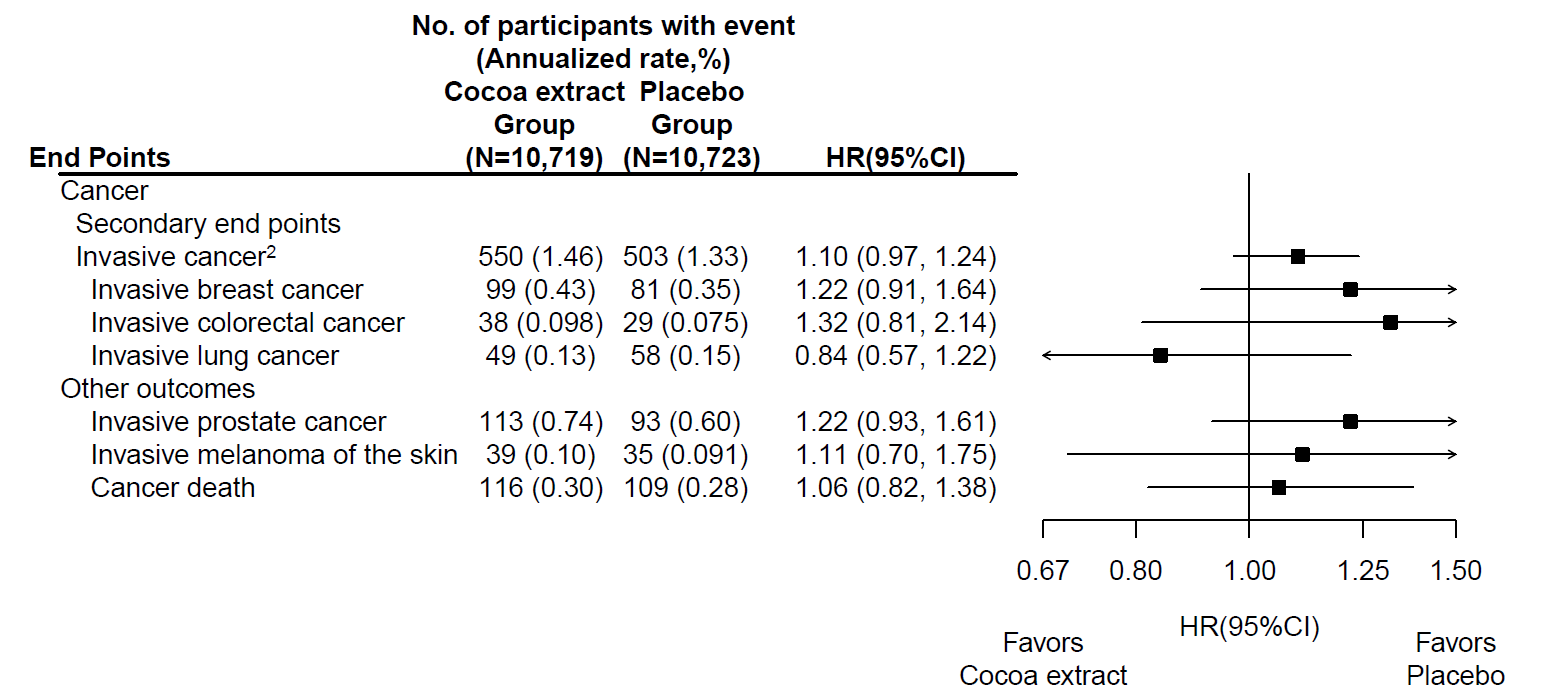


**Supplementary Figure 6. Hazard ratios and 95% confidence intervals^1^ for self-reported non-monitored outcomes^2^ according to randomized assignment, in intention-to-treat analyses.**

**­­­**


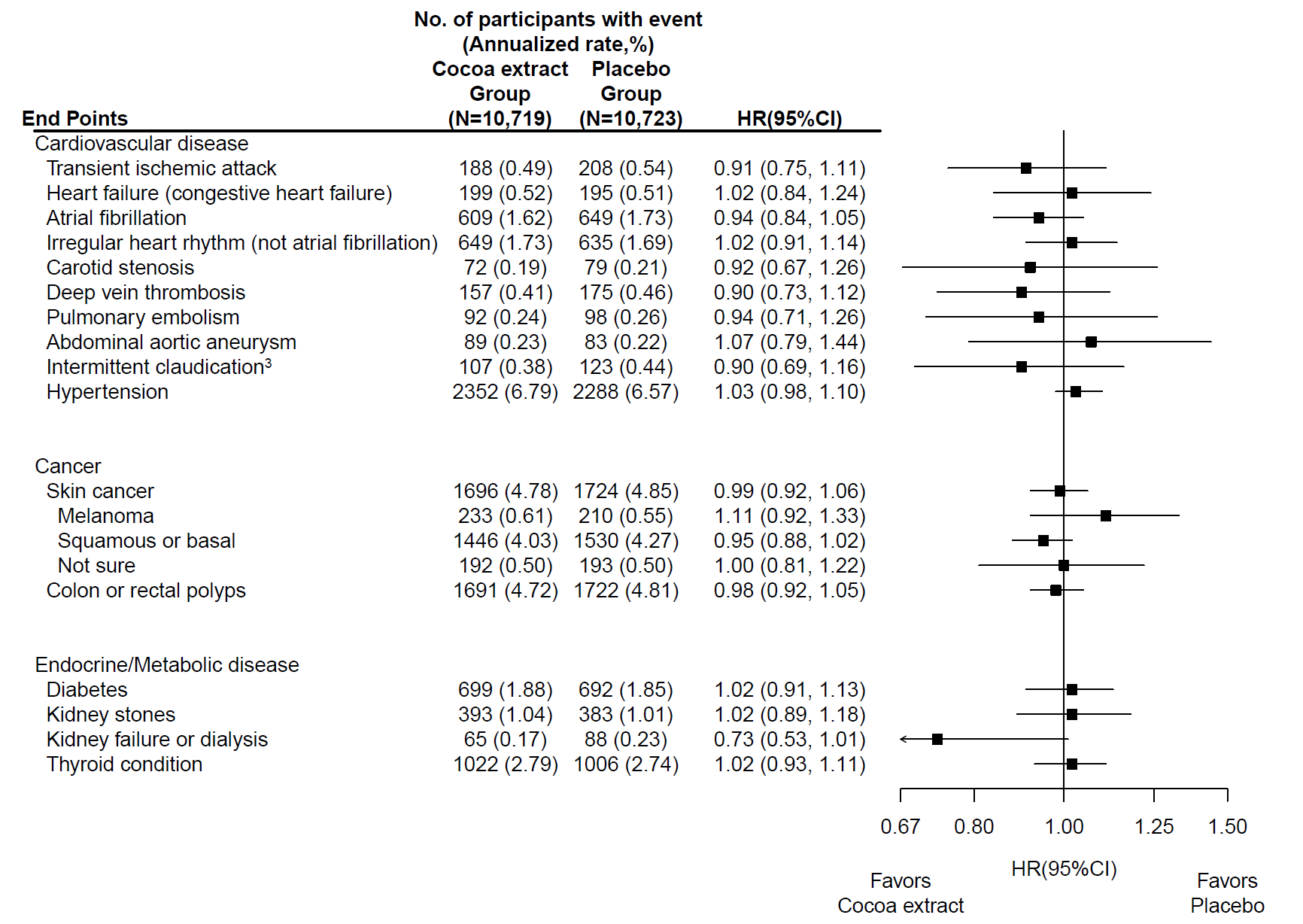


**Supplementary Figure 7. Hazard ratios and 95% confidence intervals^1^ for side effects^2^ according to randomized assignment, in intention-to-treat analyses.**


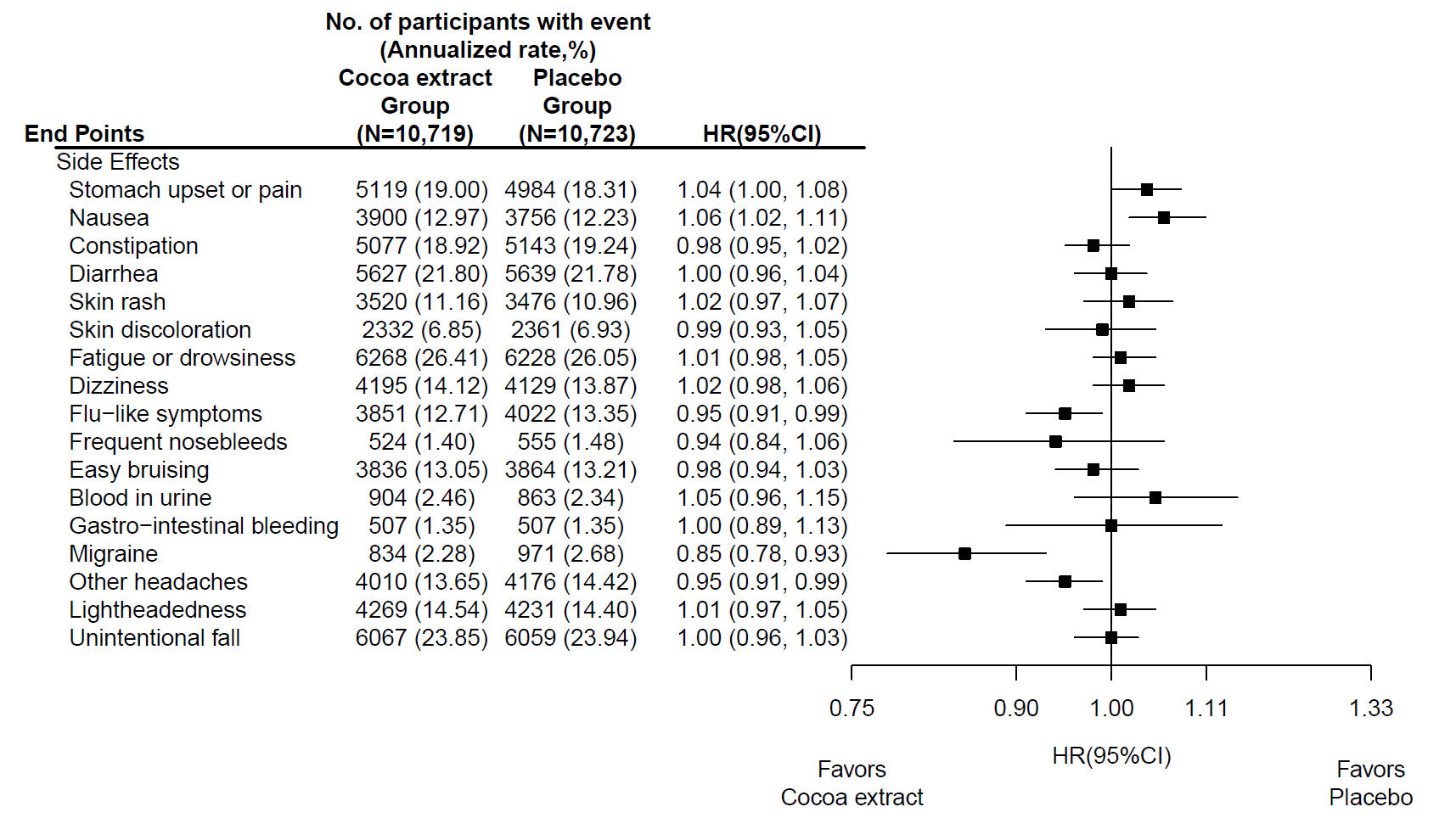

Supplement: nqac055_Supplemental_File [file nqac055_supplemental_file.docx]
